# Split-Type Multiband Filter Design Using Ultra-Miniaturized Substrate-Integrated Coaxial Cavities

**DOI:** 10.3390/mi17070814

**Published:** 2026-07-06

**Authors:** Ming-Chih Chen, Ci-Fang Jheng, Gawn-Wei Su, Chung-I G. Hsu, Min-Hua Ho

**Affiliations:** 1 AI Development Center and Graduate Institute of Business Administration, Fu Jen Catholic University, New Taipei City 242062, Taiwan; 081438@mail.fju.edu.tw; 2 Department of Electronic Engineering, National Changhua University of Education, Changhua City 50074, Taiwan; super1127968@gmail.com; 3 Wistron Corporation, Taipei 11469, Taiwan; skt297y6@gmail.com; 4 Department of Electrical Engineering, National Yunlin University of Science and Technology, Yunlin 64002, Taiwan; cghsu@yuntech.edu.tw

**Keywords:** multiband filter, split-type filter, SIW filter, substrate-integrated coaxial cavity (SICC), circuit miniaturization

## Abstract

The contribution of this paper is to propose the design and experimental validation of split-type dual- and tri-band bandpass filters (BPFs) based on highly miniaturized substrate-integrated coaxial cavities (SICCs). The proposed split-type multiband filter design achieves exceptional circuit-area efficiency within the SIW-related (substrate-integrated waveguide) split-type filter category. The size-reduced SICCs are fabricated using two substrates of different thicknesses. The coupling matrix method is employed to synthesize the responses of the example dual- and tri-band filters. The proposed dual-band filter achieves a circuit size of 0.17 *λ_d_* × 0.17 *λ_d_*, with insertion losses of 0.78 and 0.89 dB for the two passbands, and isolation between the passbands exceeding 15 dB. For the tri-band filter, the circuit size is 0.27 *λ_d_* × 0.34 *λ_d_*, with the insertion losses of 0.96, 2.6, and 1.21 dB across the three passbands, accompanied by similarly effective isolation. Experimental results validate the circuit designs and performance, demonstrating strong agreement between measured and simulated data.

## 1. Introduction

Recently, split-type multiband bandpass filters (BPFs) have attracted significant interest for use in commercial communication systems that require multifunctional and multistandard transceivers [1,2,3,4,5,6,7,8,9,10]. These split-type filters achieve dual-, triple-, or multiband responses by dividing one or two virtual passbands into two, three, or more sub-passbands, thereby realizing the desired multiband characteristics. Such multi-passband filters maintain favorable performance across all passbands, rather than only a limited subset, while providing sufficient interchannel isolation. Moreover, most wireless communication systems feature dual channels for transmitting and receiving data in one frequency band. To enhance channel efficiency, these channels typically require a very narrow guard band, which can only be effectively achieved using a split-type filter design.

Over the past two decades, two primary approaches—signal interference [11] and the well-known coupling matrix method [2,3]—have been proposed to achieve band splitting. Among these, the coupling matrix method has been more widely adopted because it divides a single passband into multiple sub-bands by introducing several transmission zeros (TZs) within a virtual wide passband. This approach significantly reduces design complexity, eliminates the need for complicated matching networks, maintains circuit miniaturization without additional auxiliary components, and mitigates the excessive insertion loss often associated with multi-stage coupling structures.

In refs. [4,5], half- and quarter-wavelength microstrip resonators were employed to realize split-type filters, in which two or three sub-passbands were generated by embedding multiple TZs within a virtual passband. To achieve more sub-passbands, additional virtual bands arising from multiple microstrip resonances are necessary. However, implementing parallel coupling among microstrip resonators is challenging because very narrow gaps are required to satisfy tight coupling conditions. Alternatively, split-type filters and diplexers based on substrate-integrated waveguides (SIWs) were reported in refs. [6,7,8,9,10]. Compared with microstrip implementations, SIWs offer superior characteristics, including higher quality factors, greater power-handling capability, and stronger immunity to environmental disturbances. These designs exploit the first few resonance modes of SIWs (TE_101_, TE_102_, and TE_201_ modes) to generate the required virtual passbands.

Despite the compact arrangement of cavities in these designs—often with no gaps between adjacent units—the lateral dimensions of each cavity are still determined by half-wavelength (TE_101_) or quarter-wavelength (TE_102_ and TE_201_) conditions. Consequently, the overall circuit size remains relatively large. Even with fractional-mode cavities or vertically stacked configurations, achieving significant size reduction remains challenging. For example, in refs. [7,8], traditional SIW cavities were used to construct split-type multiband filters by employing dual-mode resonance, including the fundamental TE_101_ mode and the higher-order TE_102_ or TE_201_ modes. In this case, the cavity’s side dimension corresponds to half the wavelength, resulting in a significantly large circuit area. The required couplings between cavities are complex, including coupling irises, coupling windows, and CPWs. In ref. [9], third-order split-type tri-band and quad-band filters were constructed using up to nine and twelve SIW cavities, respectively. The cavities were stacked to reduce the circuit size, and no cavity miniaturization techniques were applied. Coupling between the cavities was achieved through coupling slots placed on the cavity common wall. To alleviate the drawbacks of bulkiness and large circuit size in substrate-integrated waveguide (SIW) cavities, which restrict their commercial applications, various approaches have been explored [12,13,14,15,16,17,18]. Breakthroughs in SIW cavity miniaturization were achieved by introducing substantial capacitance along the cavity height, thereby transforming the SIWC into a coaxial-line cavity mode. Consequently, it is termed a substrate-integrated coaxial cavity (SICCs). In our previous studies [14,15], the circuit sizes of the proposed SICCs used in bandpass and balanced bandpass filters were reduced to 3.6% and 4.64%, respectively, of their conventional SIW cavity counterparts. Building on these results, split-type dual- and tri-band bandpass filters based on the coupling-matrix method were developed in ref. [19], thereby realizing size-reduced split-type multiband filter designs.

More recently, in refs. [20,21,22], an even smaller version of the SICC, termed the ultra-miniaturized SICC, was developed to construct conventional SICC filters, diplexers, and their balanced forms. However, these downsized SICCs still faced challenges in facilitating split-type filter design. To overcome this, we propose a size-efficient split-type dual- and tri-band filter design using these ultra-miniaturized SICCs. The proposed ultra-miniaturized SICC reduces its size to 1.9% of its conventional SIW cavity counterpart. The proposed SICC split-type multiband filter exhibits a highly area-efficient design among the SIW-related split-type filters. Experimental results are presented to validate the proposed designs, showing good agreement between measured and simulated responses.

## 2. Ultra-Miniaturized Substrate-Integrated Coaxial Cavity

**Figure 1:**
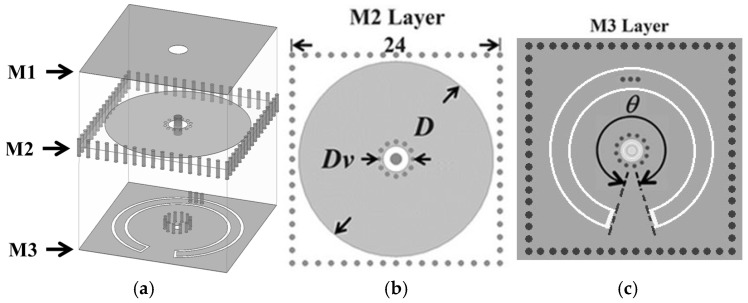
(**a**) The 3D view of the proposed SICC. (**b**) metal layer M2 (with circular patch), (**c**) metal layer M3 (consisting of embedded SCR).

As indicated in ref. [14], the SICC’s resonance frequency downshift extent primarily depends on the capacitance loaded onto the SICC. Therefore, following the approach described in ref. [20], this paper employs an ultra-miniaturized SICC with enhanced loading capacitance to design the split-type filters. Figure 1 illustrates the proposed SICC’s 3D structure and the layouts of its metal layers. It is realized using two vertically stacked RT/Duroid 5880 substrates (*ε_r_* = 2.2, tanδ = 0.0009) with different thicknesses. The thicknesses of the top and bottom substrates are 0.254 mm (denoted as *h*_1_) and 1.57 mm (denoted as *h*_2_), respectively. The SICC’s side dimension is fixed at 24 mm for easy comparison with its conventional SIW cavity counterpart in ref. [23] and the size-reduced SICCs used in refs. [14,15,19]. The miniaturization of the proposed SICC depends on the two integrated capacitances. One capacitance is formed by the circular patch (shown in Figure 1b) together with the cavity’s top and bottom walls (i.e., the M1 and M3 layers in Figure 1a). This capacitance, denoted as *C_d_*, is evaluated by $C_{d}\approx 0.25\pi \varepsilon D^{2}(1/h_{1}+1/h_{2})$. The other capacitance is facilitated by the split coplanar-waveguide ring (SCR) embedded in the M3 layer (shown in Figure 1c). The notation, *θ*, is the SCR’s subtended angle. The SCR is connected to the circular patch through three buried via holes, resulting in the two capacitances being configured in a shunt connection. In practice, the cavity is excited by an SMA connector whose central probe penetrates the cavity and attaches to the bottom wall.

**Figure 2:**
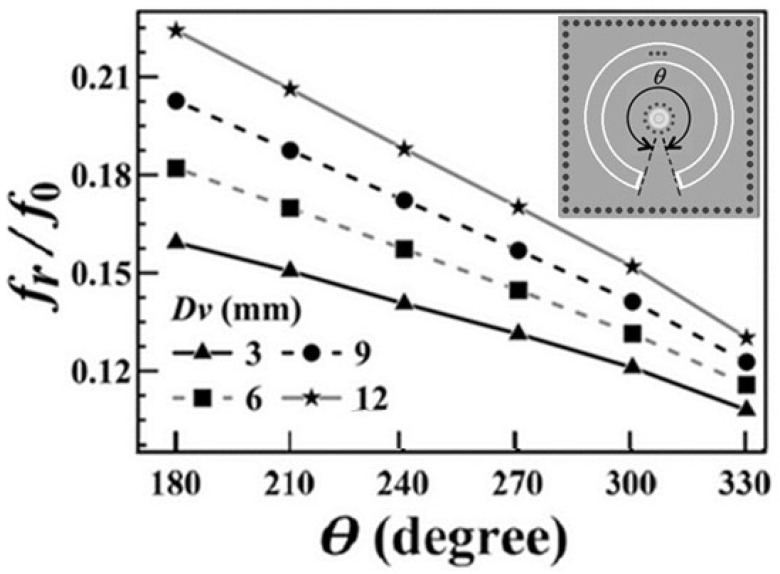
The computed *f_r_*/*f*_0_ versus the embedded SCR’s subtended angle, *θ*, for the SICC in Figure 1 with several *D_v_* values.

Referring to ref. [20] without delving into details in this paper, the computed curves of the proposed SICC’s normalized resonance frequency (*f_r_*/*f*_0_) are provided in Figure 2. Here, the *f*_0_ representing the resonance frequency of the SIW cavity counterpart is 5.96 GHz. The typical dimensions used in the calculations are as follows: circular patch diameter *D* = 22.5 mm; SCR outer diameter is 20.8 mm; widths of the CPW’s central strip and gaps are 1.35 mm and 0.2 mm, respectively. In Figure 2, the lowest frequency ratio is approximately 0.12, occurring at the maximum dimension parameters of *D_v_* = 3 mm and *θ* = 330°. It corresponds to an ultimate area-reduction ratio (defined as (*f_r_*/*f*_0_)^2^) of only 1.44%.

**Figure 3:**
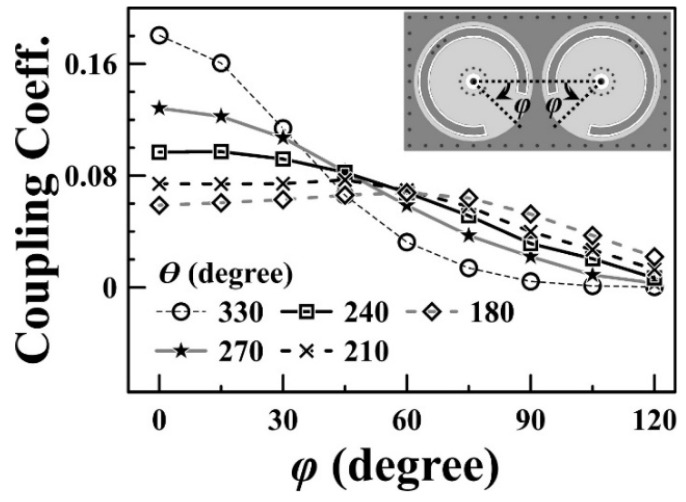
Calculated curve of the coupling coefficients versus the rotation angle (*φ*) of the SCR’s split gaps with several values of *θ*.

The coupling coefficient between the two adjacent SICCs is first discussed in ref. [20]. For convenience, Figure 3 presents the coefficient as a function of the rotation angle (*φ*) of the SCRs. Note that the coupling coefficient is inversely proportional to the rotation angle (*φ*), where *φ* = 0° indicates that the split gaps of the two SCRs directly face each other, resulting in maximum coupling. The rotations of the two SCRs are clockwise for the left SCR and counterclockwise for the right SCR. The circuit parameters used in the calculation are: *Dv* = 4 mm; the rest are the same as in Figure 2. As illustrated in Figure 3, the coupling strength between cavities primarily depends on the rotation angle (*φ*) of the SCRs and their subtended angles (*θ*). Iterative simulations are employed to determine and refine the parameters of the SICCs, starting from appropriate initial values. Specifically, critical dimensions such as the rotation angle (*φ*) and the subtended angles (*θ*) of the SCRs are determined and refined through extensive simulations, aided by the matrix from CMS. Additionally, the effects of the coupling coefficients on the sub-passbands—including sub-passband bandwidth control, guard-band bandwidth, and the frequency separations between sub-passbands—were thoroughly illustrated in our previous work [19].

## 3. Sample Results and Performance Comparison

**Figure 4:**
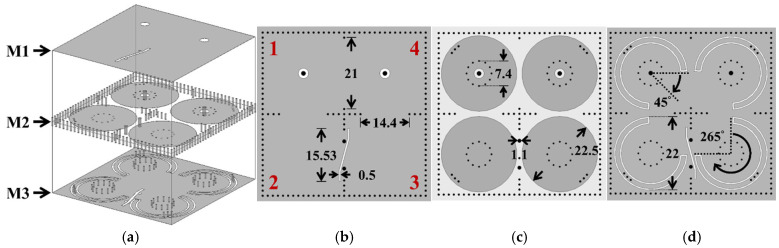
Structure of the proposed SICC split-type dual-band filter (denoted as Filter I). (**a**) 3D view, (**b**) M1, (**c**) M2, and (**d**) M3 layouts and their circuit dimensions (unit: mm).

Figure 4 illustrates the structure and circuit dimensions of the proposed SICC split-type dual-band filter. For clarity, this dual-band filter is referred to as Filter (I). The filter consists of four SICCs, as described in Section 2, with the cavity numbers labeled at the corners of the third metal layer. The natural resonance frequency of the employed SICC is approximately 0.96 GHz, making the area of a single SICC only 2.6% that of a conventional SIW cavity operating at the same frequency. Cavities 1 and 3 are excited, while the others serve as loaded cavities. Couplings between cavities (1, 2), (1, 4), and (3, 4) are dominated by the magnetic field and are termed positive coupling. In contrast, the coupling between cavities (2, 3) is primarily governed by the electric field and is therefore termed negative coupling. Adopted from ref. [24], this negative coupling is achieved through a complementary set of bent slots located on the top and bottom walls along the common boundary of the cavities, combined with a pair of through-via holes positioned between the slots.

**Figure 5:**
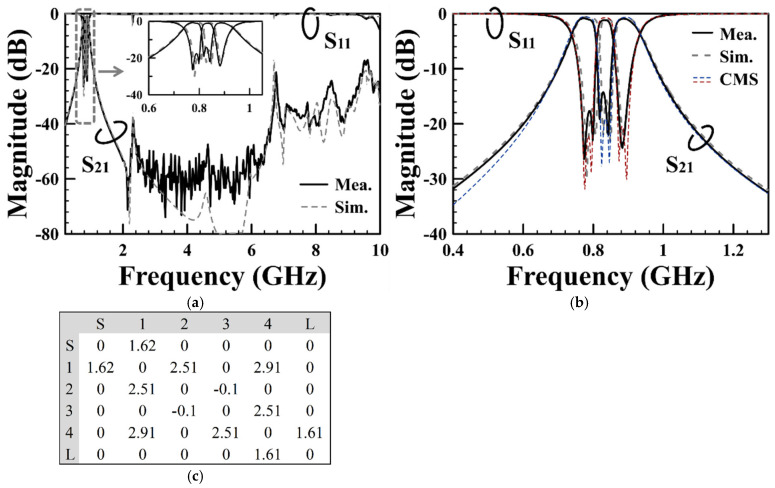
Measured and simulated frequency responses (CMS and HFSS) of the Filter I shown in Figure 4: (**a**) wideband response, (**b**) zoomed-in passband response, and (**c**) the coupling matrix used in CMS simulation.

**Figure 6:**
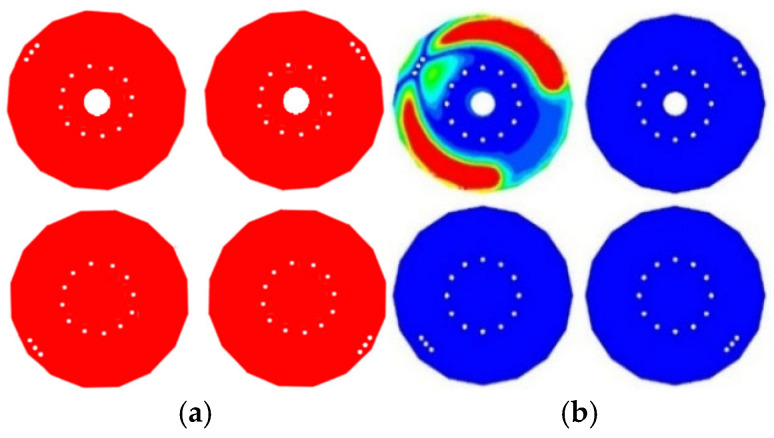
Simulated E-field distributions on the circular patch at (**a**) the in-band frequency (0.78 GHz) and at (**b**) the rejection band frequency (2.42 GHz).

Figure 5 compares the measured and simulated frequency responses obtained from electromagnetic (EM) simulation software, including ANSYS HFSS [25] and coupling matrix synthesis (CMS) [26]. The coupling matrix shown in Figure 5c is used in the CMS simulation. The figure presents both responses in a wideband scope and a zoomed-in view of the passband. Simulated electric field (E-field) distributions on the circular patch of the dual-band filter are presented in Figure 6 at the in-band frequency (0.78 GHz), illustrating the operating mode of the SICCs, as well as the higher-order mode at the rejection-band frequency (2.42 GHz). The effects of the coupling coefficients on the sub-passbands—such as sub-passband bandwidth control, guard-band bandwidth, and the frequency separations between the sub-passbands—have been thoroughly discussed in ref. [19]. For example, the coupling between cavities (1, 4) dominates the passband bandwidth, while the separation of passbands is simultaneously regulated by the couplings between cavities (1, 2) and cavities (3, 4). The experimental circuit was measured using an Agilent E8361C (Agilent Technologies, Santa Clara, CA, USA) vector network analyzer (VNA) with a frequency step of 1 MHz. The TRL calibration procedure was employed, which also de-embedded the effects of the SMA connectors. The measured and simulated circuit parameters—including the sub-band mid frequencies (*f_i_*, *I* = 1, 2), 3 dB fractional bandwidths (FBWs), in-band minimum insertion losses (ILs), frequencies of implanted transmission zeros (TZs), and upper stopband bandwidth—are listed in Table 1. Measurement tolerances from fabrication are negligible because the PCB manufacturing process ensures precise layer alignment and accurate circuit layouts. The periodicity of the via-hole arrays forming the cavity boundary is maintained at less than one-twentieth of the guided (or intrinsic) wavelength, effectively confining the E-field within the cavity.

**Figure 7:**
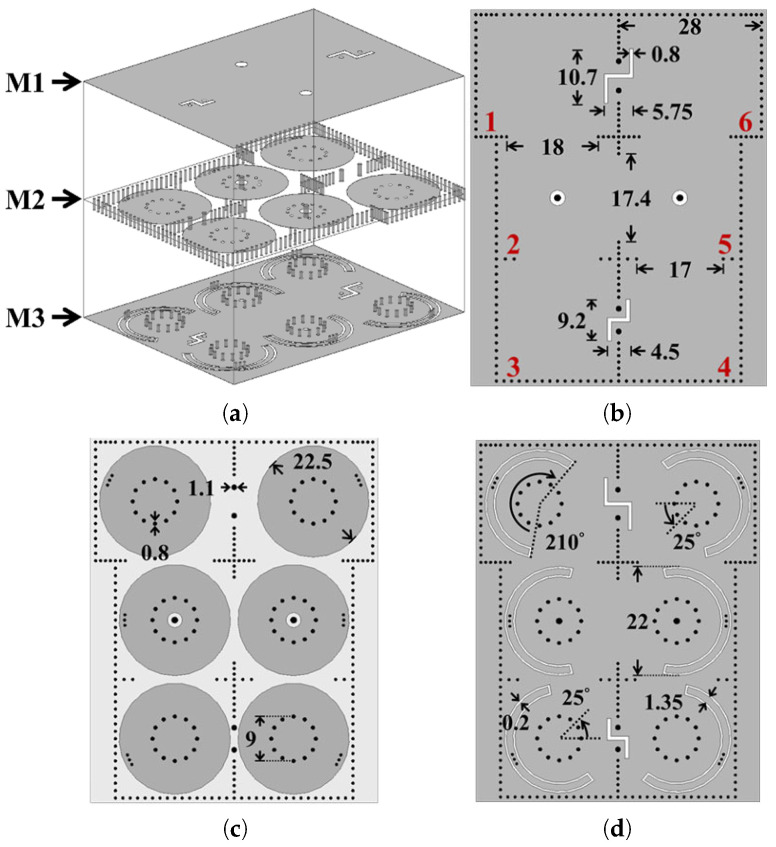
Structure of the proposed SICC split-type tri-band filter (Filter II). (**a**) 3D view, (**b**) M1, (**c**) M2, and (**d**) M3 layouts and their circuit dimensions (unit: mm).

**Figure 8:**
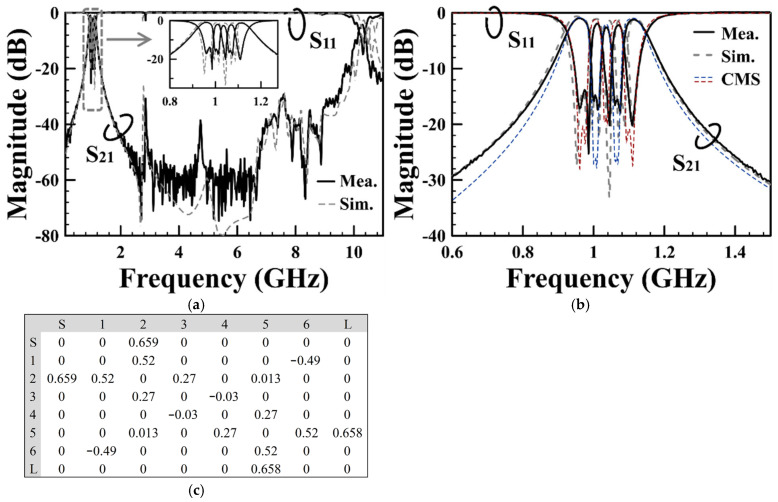
Measured and simulated frequency responses of the Filter II in Figure 7: (**a**) wideband scope, (**b**) zoomed-in passband, and (**c**) the coupling matrix for CMS simulation.

Figure 7 presents the structure, circuit layouts, and dimensions of the proposed SICC split-type tri-band filter (denoted as Filter II). The filter uses six SICCs; most adjacent cavities are magnetically coupled, except for cavities (1, 6) and (3, 4), which are electrically coupled. The input/output SMA feeds are connected to the middle two cavities (i.e., cavities 2 and 5 in Figure 7b), while the upper and lower cavities serve as loaded cavities, providing alternative signal routes. The SICC’s natural resonance frequency is around 1.14 GHz, resulting in the SICC’s area being only 3.7% of that of a conventional SIW cavity operating at the same frequency. Figure 8 shows the measured and simulated frequency responses obtained using CMS and HFSS simulations, including both wideband and passband zoomed-in views, as well as the coupling matrix required for CMS simulation.

**Figure 9:**
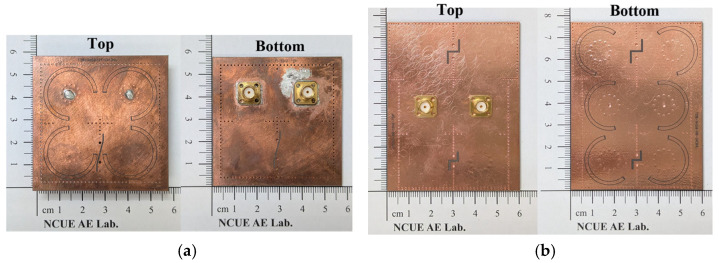
The photos of the experimental circuits for (**a**) Filter I and (**b**) Filter II.

The measured and simulated circuit parameters—including the sub-bands’ mid-band frequencies (*f_i_*, *I* = 1, 2, 3), 3 dB fractional bandwidths (FBWs), in-band minimum insertion losses (Ils), and upper stopband BW—are listed in Table 2. The measured excessive loss of 2.6 dB in the second passband, compared to the simulated 1.42 dB, might be attributed to coupling tolerances arising from actual circuit fabrication. However, the insertion losses in the other two sub-passbands are less sensitive to variations in coupling. Shown in Figure 9 are photographs of Filters I and II fabricated for the measurements. For comparison, our design and several other SIW-related split-type filters are listed in Table 3. The table presents circuit parameters, including the sub-bands’ mid-band frequencies, FBWs, Ils, number of cavities, circuit size (in terms of *λ_d_*), and the upper stopband BW. Note that *λ_d_* in Table 3 represents the intrinsic wavelength within the dielectric medium at the center frequency of the first passband.

## 4. Conclusions

This paper presents the design and experimental validation of SICC split-type dual- and tri-band filters based on ultra-miniaturized substrate-integrated coaxial cavities (SICCs). The proposed dual- and tri-band filter’s circuit area occupies only 0.17 *λ_d_* × 0.17 *λ_d_* and 0.27 *λ_d_* × 0.34 *λ_d_* of the circuit area, respectively. This design demonstrates exceptional efficiency in circuit area utilization. Additionally, low passband insertion losses are achieved across all operating bands. The close agreement between measured and simulated results further validates the effectiveness of the proposed filter designs.

## Figures and Tables

**Table 1:** Measured and simulated circuit parameters for Filter I.

	Simulations	Measurements
*f*_1_/*f*_2_ (GHz)	0.78/0.894	0.77/0.89
3 dB FBWs (%)	8.97/6.71	7.78/7.78
Minimum Ils (dB)	0.58/0.68	0.8/0.89
TZs (GHz)	0.83 & 0.85	0.82 & 0.84
USBW (|S_21_| ≤ −20 dB)	10.9 *f*_1_	10.88 *f*_1_

USBW: abbreviation for upper stopband BW.

**Table 2:** Measured and simulated circuit parameters for Filter II.

	Simulations	Measurements
*f*_1_/*f*_2_/*f*_3_ (GHz)	0.964/1.04/1.12	0.96/1.03/1.12
3 dB FBWs (%)	7.26/2.87/4.47	6.22/1.36/4.27
Minimum ILs (dB)	0.64/1.42/0.94	0.96/2.6/1.21
TZs (GHz)	1.01/1.02 & 1.07/1.08	1.0/1.01 & 1.06/1.08
USBW (|S_21_| ≤ −20 dB)	8.96 *f*_1_	8.72 *f*_1_

**Table 3:** Performance comparison for our works and SIW-related split-type filter references.

	*f*_1_/*f*_2_/*f*_3_/*f*_4_ (GHz)	FBWs (%)	ILs (dB)	Cavity No.	Size (*λ_d_* × *λ_d_*)	USBW (|S_21_| ≤ −20 dB)
[7] Figure 4	11.56/12.45/ 13.53/14.36	4.4/3.29/ 2.72/3.25	0.89/1.27/ 1.45/1.36	4	1.52 × 1.1	~0.07 *f*_1_
[8] Figure 5	12/13.34/13.92	3.0/2.25/2.44	2.48/1.71/1.82	6	2.84 × 1.7	~0.084 *f*_1_
[9] Filter I	4.81/5.08/5.35	4.48/1.84/4.71	1.41/2.64/1.39	9	1.18 × 0.58	NA
[9] Filter II	4.67/4.91/ 5.09/5.34	3.63/1.91/ 1.61/3.91	1.64/2.66/ 2.96/1.87	12	1.18 × 0.61	NA
[19] Figure 4	1.82/2.03	3.85/3.45	1.73/1.28	4	0.43 × 0.43	4.84 *f*_1_
[19] Figure 9	1.69/1.8/1.91	3.08/2.55/3.02	1.12/1.32/1.11	6	0.5 × 0.6	4.72 *f*_1_
Filter I	0.77/0.89	7.78/7.78	0.8/0.89	4	0.17 × 0.17	10.88 *f*_1_
Filter II	0.96/1.03/1.12	6.22/1.36/4.27	0.96/2.6/1.21	6	0.27 × 0.34	8.72 *f*_1_

## Data Availability

The data presented in this study are available on request from the corresponding author.
